# CHAC1 degradation of glutathione enhances cystine-starvation-induced necroptosis and ferroptosis in human triple negative breast cancer cells via the GCN2-eIF2α-ATF4 pathway

**DOI:** 10.18632/oncotarget.23055

**Published:** 2017-12-09

**Authors:** Meng-Shian Chen, Sheng-Fan Wang, Chih-Yi Hsu, Pen-Hui Yin, Tien-Shun Yeh, Hsin-Chen Lee, Ling-Ming Tseng

**Affiliations:** ^1^ Department and Institute of Pharmacology, School of Medicine, National Yang-Ming University, Taipei 112, Taiwan; ^2^ Taipei-Veterans General Hospital, Comprehensive Breast Health Center, Taipei 112, Taiwan; ^3^ Department of Pharmacy, Taipei Veterans General Hospital, Taipei 112, Taiwan; ^4^ Department of Pathology and Laboratory Medicine, Taipei Veterans General Hospital, Taipei 112, Taiwan; ^5^ Department of Medical Research, Taipei Veterans General Hospital, Taipei 112, Taiwan; ^6^ Department of Anatomy and Cell Biology, School of Medicine, National Yang-Ming University, Taipei 112, Taiwan; ^7^ Department of Surgery, Taipei Veterans General Hospital, Taipei 112, Taiwan; ^8^ Department of Surgery, School of Medicine, National Yang-Ming University, Taipei 112, Taiwan

**Keywords:** TNBC, cystine starvation, glutathione, necroptosis, ferroptosis

## Abstract

Cancer cells exhibit an abnormal amino acid metabolism and a dependence on specific amino acids, which might provide potential targets for treating cancer patients. In this study, we demonstrated that human triple negative breast cancer (TNBC) cells were highly susceptible to cystine starvation. We found that necrostatin-1 (Nec-1, a RIP1 inhibitor), necrosulfonamide (an MLKL inhibitor), deferoxamine (an ion chelator), ferrostatin-1 (a ferroptosis inhibitor) and RIP1 knockdown can prevent cystine-starvation-induced cell death, suggesting that cystine starvation induces necroptosis and ferroptosis in TNBC cells. Moreover, cystine starvation induced mitochondrial fragmentation, dysfunction, and ROS production. A mitochondrial ROS scavenger, Necrox-5, can prevent cystine-starvation-induced cell death. In addition, cystine starvation was found to activate GCN2, but not PERK, to increase the phosphorylation of eIF2α at serine 51, the protein expression of ATF4, and the expression of ATF4 target genes such as CHAC1, which might be downstream of the RIP1/RIP3-MLKL pathway and contribute to cystine-starvation-induced cell death. Knockdown of CHAC1 rescued the cystine-starvation-induced reduction in glutathione (GSH) levels and cell death. Furthermore, N-acetyl-cysteine (NAC), Trolox, and Nec-1 significantly prevented the cystine-starvation-induced increase in intracellular ROS levels, mitochondrial fragmentation and cell death. In summary, these results suggest that CHAC1 degradation of GSH enhances cystine-starvation-induced necroptosis and ferroptosis through the activated GCN2-eIF2α-ATF4 pathway in TNBC cells. Our findings improve our understanding of the mechanism underlying cystine-starvation-induced TNBC cell death.

## INTRODUCTION

Breast cancer is one of the leading causes of cancer-related death in females. The triple-negative breast cancer (TNBC) subgroup, which is estrogen-receptor (ER) negative, progesterone-receptor (PR) negative, and HER-2 negative, accounts for approximately 15–18% of breast cancers [1, 2]. TNBC has an aggressive behavior and a lack of targeted therapies, which results in chemotherapy as the main treatment and a poor prognosis [1, 3]. Improving the outcome in this subtype requires a better understanding of the biology and the identification of new drug targets.

Cancer cells frequently exhibit metabolic changes towards anabolic metabolism and maintenance of the reduction-oxidation (redox) balance, which promotes cancer cell proliferation and enables cancer cells to adapt to or overcome stresses [4]. These cancer-associated metabolic rearrangements have been linked to the activation of proto-oncogenes and to the inactivation of tumor suppressor genes [4]. Moreover, cancer cells may increase the uptake of and reliance on environmental nutrients, such as glucose [5] or certain amino acids [6–12]. Cancer-associated amino acid dependence is associated with a specific enzyme deficiency or the elevated expression of transporters [7, 9, 10, 13]. Once nutrient deprivation occurs, cancer cells are susceptible to death. Thus, targeting cancer metabolism has been proposed as a promising strategy for the development of anti-cancer therapy [4].

Recently, an analysis of glutamine sensitivity identified the Xc- cystine/glutamate antiporter as a common TNBC therapeutic target [13]. This plasma membrane transporter mediates the cellular uptake of cystine from the environment in exchange for intracellular glutamate [14]. This transporter is known to contribute to the maintenance of intracellular glutathione (GSH) levels and the protection of cells from oxidative stress and is essential for proliferation, therapy resistance and progression in certain cancer cells [13, 15–17]. These findings implied that TNBC cells seem to highly depend on an extracellular source of amino acids for cell survival and proliferation. However, it is still unclear whether a specific amino acid is essential for TNBC cell growth.

Nutrient starvation has been reported to induce different types of cell death, including apoptosis [18], necroptosis [9], non-apoptotic, iron-dependent, oxidative death (ferroptosis) [19], and autophagy-mediated cell death [20]. However, the death mechanism seems to be caused by different extents of starvation of the same nutrient or different nutrients, or by different cell genotypes. In addition, in response to diverse stress stimuli, such as glucose deprivation and amino acid deprivation, eukaryotic cells activate a common integrated stress response (ISR) to adapt to the stress condition or to restore cellular homeostasis [21]. If the cellular stress is severe, the ISR pathway will become activated to execute cell death [21]. However, it is not clear whether or how the ISR pathway induces different types of cell death in TNBC cells in response to specific nutrient starvation.

In this study, we first screened for the amino acid that is essential for TNBC cell growth, evaluated the type of cell death induced by starvation of that amino acid, and dissected the molecular mechanism responsible for amino-acid-starvation-induced cell death in TNBC cells.

## RESULTS

### Cystine starvation induces necroptosis and ferroptosis in TNBC cells

**Figure 1:**
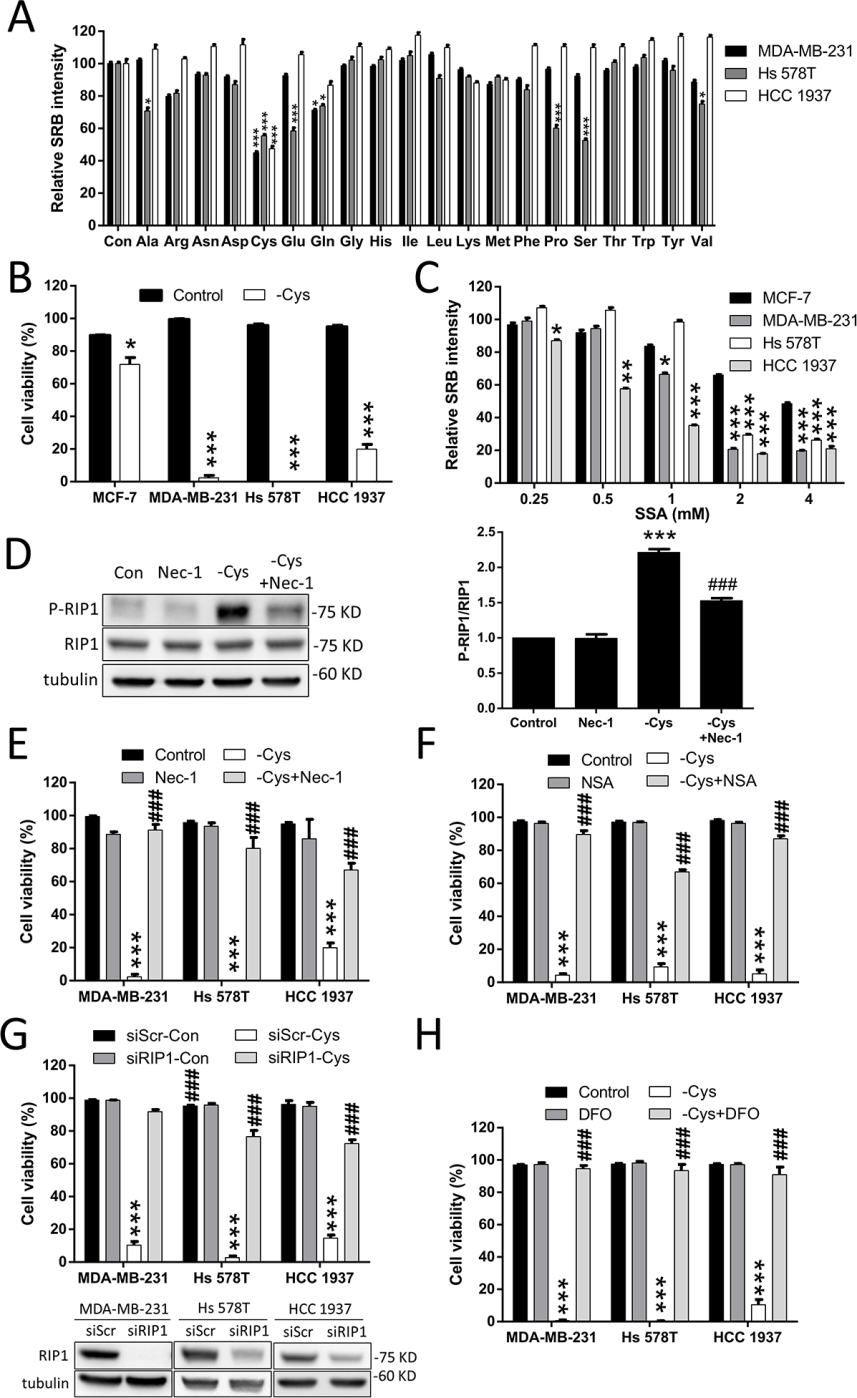
Cystine starvation induces necroptosis and ferroptosis in TNBC cells (**A**) Human breast cancer cells (MDA-MB-231, Hs 578T, and HCC 1937) were treated with the 20 amino acid starvation mediums for 48 h. Cell growth was determined by SRB. (**B**) The breast cancer cells (MCF7, MDA-MB-231, Hs 578T, and HCC 1937) were treated with cystine starvation for 48 h. Cell viability was determined using the trypan blue exclusion assay. (**C**) The breast cancer cells were treated with different concentrations of SSA for 72 h. The relative number of viable cells was determined using the SRB assay. (**D**) Hs 578T cells were treated with cystine starvation with or without 16 μM Nec-1 for 9 h. The P-RIP1 and RIP1 levels were determined using Western blotting. (**E**, **F**, **G**, **H**) The breast cancer cells (MDA-MB-231, Hs 578T, and HCC 1937) were treated with cystine starvation with or without 16 μM Nec-1 (E), 20 μM NSA (F), RIP1 siRNA (G), or 100 μM DFO (H) for 48 h. Cell viability was determined using the trypan blue exclusion assay. The knockdown efficiency of RIP1 was detected using Western blotting. Data represent the mean ± SEM of three independent experiments. ^*^p < 0.05, ^**^p < 0.01, ^***^p < 0.001 compared to the control group; ^#^*p* < 0.05, ^##^*p* < 0.01, ^###^*p* < 0.001 compared to the cystine starvation group. Con, control; -Cys, cystine starvation; SSA, sulfasalazine; Nec-1, necrostatin-1; NSA, necrosulfonamide; DFO, deferoxamine.

To identify which amino acid is important for TNBC cell growth, we grew three human TNBC cell lines, MDA-MB-231, Hs 578T, and HCC 1937, in different amino acid starvation media for 48 h. Figure 1A shows that cystine starvation significantly reduces the cell number. Using the trypan blue exclusion assay, we confirmed that cystine starvation induces cell death in all three TNBC cell lines but not in the estrogen-receptor-positive MCF-7 cell line (Figure 1B). We further confirmed the results by flow cytometry with propidium iodide (PI) exclusion assay (Supplementary Figure 1A). Moreover, we treated these breast cancer cells with the Xc- cystine/glutamate antiporter inhibitor sulfasalazine (SSA) and found that the three TNBC cell lines are more sensitive to sulfasalazine treatments than the MCF-7 cell line (Figure 1C). These results suggest that cystine is required for cell growth and that cystine starvation induces cell death in these TNBC cell lines.

Nutrient starvation has been reported to induce different types of cell death. Based on the previous reports that inhibition of this Xc- cystine/glutamate antiporter induces necroptosis [9] or ferroptosis [22–24] in different cancer cell types, we first evaluated whether the cystine starvation-induced cell death in TNBC cells is through necroptosis or ferroptosis.

Necroptosis is a form of programmed necrosis, which is regulated by Receptor-Interacting Protein 1 (RIP1), RIP3, and Mixed Lineage Kinase Domain-Like (MLKL). Upon activation, RIP1 and RIP3 bind to each other to form necrosome and promote RIP3 auto-phosphorylation and subsequent activation, allowing RIP3 to recruit and phosphorylate MLKL. This results in oligomerization of MLKL, membrane insertion of MLKL oligomers, disruption of plasma membrane integrity, and necroptotic death [25, 26]. Therefore, RIP1, RIP3 and MLKL serve as specific markers of necroptotic death. Activation of RIP1, RIP3, and MLKL in necroptosis can be detected by changes in their phosphorylation status or membrane accumulation using immunoblotting [27, 28].

In the treatment with cystine starvation, we found that the phosphorylation of RIP1 at serine 166 is increased and that co-treatment with necrostatin-1 (Nec-1, a RIP1 inhibitor) prevents the cystine-starvation-induced RIP1 phosphorylation (Figure 1D). Moreover, treatment with Nec-1 (Figure 1E) and necrosulfonamide (NSA, a MLKL inhibitor) (Figure 1F) and the knockdown of RIP1 with siRNA against RIP1 (Figure 1G) can prevent cystine-starvation-induced cell death. We further confirmed the results by flow cytometry with PI exclusion assay (Supplementary Figure 1A). These results indicate that cystine starvation may induce necroptosis in these TNBC cells. In addition, the iron chelator deferoxamine (DFO) and ferrostatin-1 (a ferroptosis inhibitor) can significantly inhibit cystine-starvation-induced cell death (Figure 1H and Supplementary Figure 1). These results suggest that cystine starvation induces necroptosis and ferroptosis in these TNBC cells.

### Apoptosis and autophagy-mediated cell death are not involved in cystine-starvation-induced cell death

**Figure 2:**
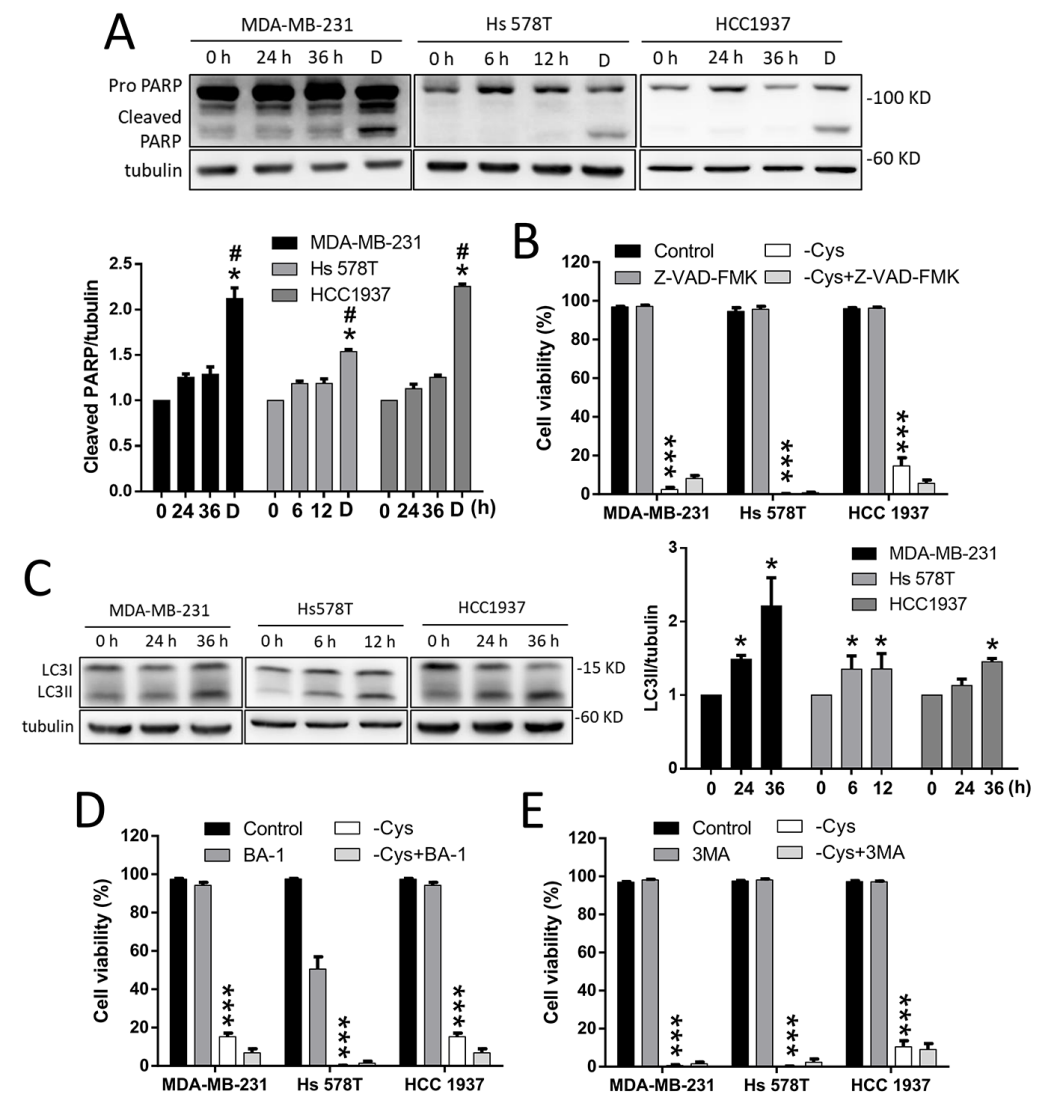
Apoptosis and autophagy-dependent cell death are not involved in cystine-starvation-induced cell death (**A**) MDA-MB-231 and HCC 1937 cells were treated with cystine starvation for 24 and 36 h, and Hs 578T cells were treated with cystine starvation for 6 and 12 h. The cleaved PARP levels were determined using Western blotting. The breast cancer cells were treated with 2 μM doxorubicin for 24 h as a positive control for the cleaved PARP. (**B**) The breast cancer cells (MCF7, MDA-MB-231, Hs 578T, and HCC 1937) were treated with cystine starvation with or without 50 nM pan-caspase inhibitor (Z-VAD-FMK) for 48 h. Cell viability was determined using the trypan blue exclusion assay. (**C**) MDA-MB-231 and HCC 1937 cells were treated with cystine starvation for 24 and 36 h, and Hs 578T cells were treated with cystine starvation for 6 and 12 h. The LC3II levels were determined using Western blotting. **(D**, **E**) The breast cancer cells (MDA-MB-231, Hs 578T, and HCC 1937) were treated with cystine starvation with or without either 50 nM BA-1 (D) or 50 μM 3-MA (E) for 48 h. Cell viability was determined using the trypan blue exclusion assay. Data represent the mean ± SEM of three independent experiments. ^*^p < 0.05, ^**^p < 0.01, ^***^p < 0.001 compared to the control group; ^#^*p* < 0.05, ^##^*p* < 0.01, ^###^*p* < 0.001 compared to the cystine starvation group. Con, control; -Cys, cystine starvation; D, doxorubicin; 3-MA, 3-Methyladenine; BA-1, bafilomycin A1.

**Figure 3:**
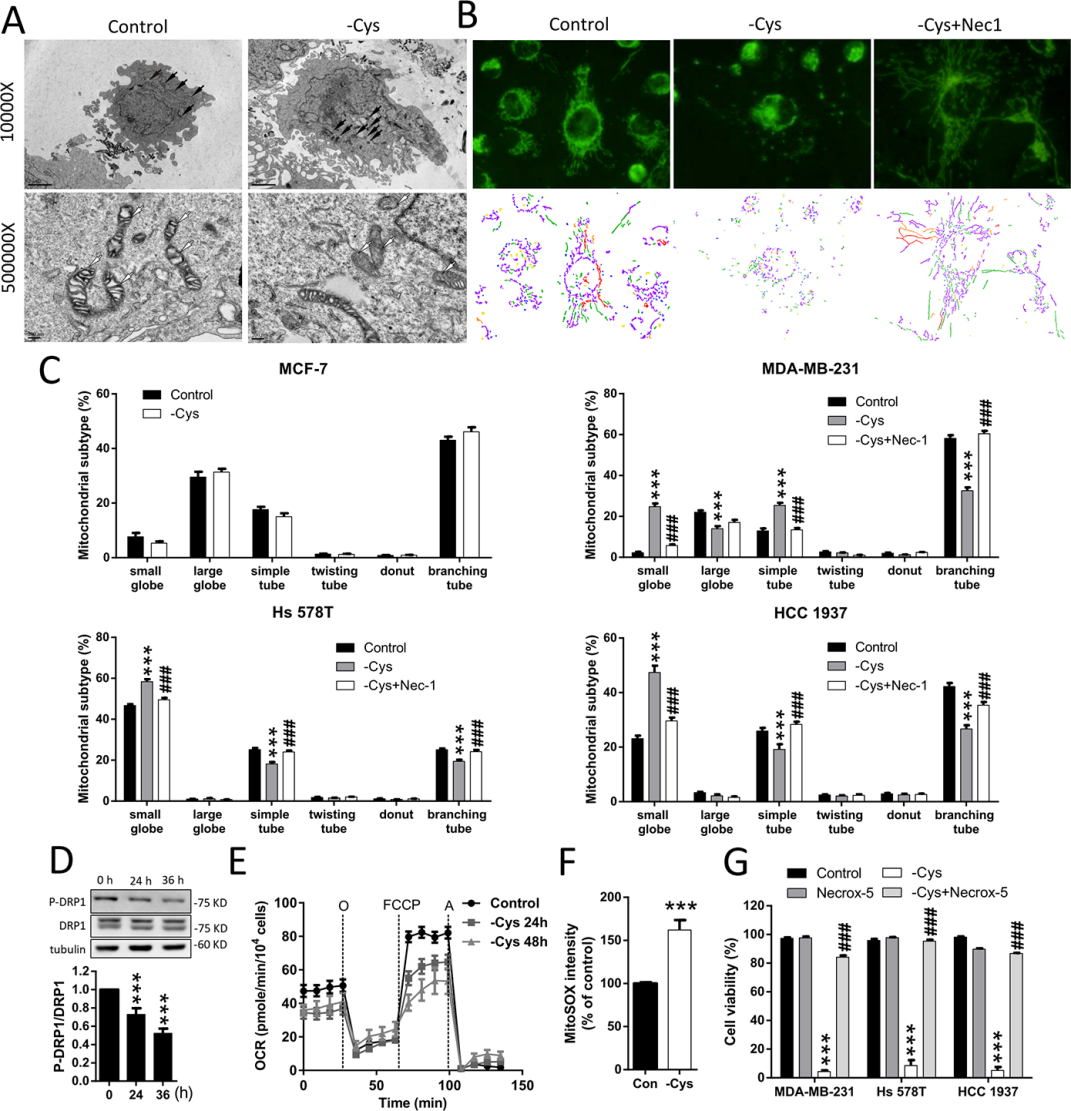
Cystine starvation induces mitochondrial fragmentation, dysfunction, and ROS production (**A**) MDA-MB-231 cells were treated with cystine starvation for 24 h. The mitochondrial morphology was determined by an EM-2000EXII transmission electron microscope. Black arrows, mitochondrial morphology; white arrows, cristae structure. (**B**, **C**) MCF-7, MDA-MB-231, Hs 578T, and HCC 1937 cells were treated with cystine starvation with or without 16 μM Nec-1 for 24 h. The mitochondrial morphology was determined using fluorescence microscopy with MitoTracker Green staining, and the mitochondrial morphology subtypes were classified by MicroP software (small globe, blue; large globe, yellow; simple tube, green; twisting tube, orange; donut, red; branching tube, purple). (**D**) MDA-MB-231 cells were cystine-starved for 24 and 36 h. The P-DRP1 (serine 637) and DRP1 levels were determined using Western blotting. (**E**) MDA-MB-231 cells were treated with cystine starvation for 24 and 48 h. The oxygen consumption rate was determined using an XF24 Extracellular Flux Analyzer. (**F**) MDA-MB-231 cells were treated with cystine starvation for 24 h. Mitochondrial ROS was determined using flow cytometry with MitoSOX red staining. (**G**) MDA-MB-231, Hs 578T, and HCC 1937 cells were treated with cystine starvation with or without 5 μM Necrox-5 for 48 h. Cell viability was determined using the trypan blue exclusion assay. Data represent the mean ± SEM of three independent experiments. ^*^p < 0.05, ^**^p < 0.01, ^***^p < 0.001 compared to the control group; ^#^*p* < 0.05, ^##^*p* < 0.01, ^###^*p* < 0.001 compared to the cystine starvation group. Con, control; -Cys, cystine starvation; Nec-1, necrostatin-1; Necrox-5, methanesulfonate.

We further examined whether apoptosis or autophagy is involved in cystine-starvation-induced cell death in TNBC cells. The results revealed that the cleaved form of PARP is not increased by cystine starvation (Figure 2A). Moreover, a pan-caspase inhibitor (Z-VAD-FMK) was not able to prevent cystine-starvation-induced cell death (Figure 2B). Moreover, although LC3II is found to be significantly increased in these TNBC cells under cystine starvation (Figure 2C), treatment with the autophagy inhibitors bafilomycin A1 (BA-1, Figure 2D) and 3-methyladenine (3-MA, Figure 2E) were not able to prevent cystine-induced cell death. These results suggest that apoptosis and autophagy-mediated cell death might not be involved in cystine-starvation-induced cell death in these TNBC cells.

### Cystine starvation induces mitochondrial fragmentation, dysfunction, and ROS production

The RIP1/RIP3/MLKL signaling in necroptosis has been linked to mitochondrial fragmentation, which is mediated by the phosphoglycerate mutase family member 5 (PGAM5)-regulated dephosphorylation of serine 637 or phosphorylation of serine 616 of dynamin-related protein (DRP1) [27]. Using transmission electron microscopy (TEM), we noted that small mitochondria with a loss of cristae structure are more frequently observed in the MDA-MB-231 cells under cystine starvation compared with the control cells (Figure 3A). To confirm this finding, we stained mitochondria with MitoTracker Green and observed the mitochondrial morphology by fluorescence microscopy (Figure 3B). We found that cystine starvation induces mitochondrial fragmentation. Quantitative results revealed that cystine starvation increases the proportion of small-globe type of mitochondria and decreases the proportions of branching-tube type of mitochondria in the three TNBC cell lines, but there are no significant changes in MCF-7 cells (Figure 3C). These results suggest that cystine starvation induces mitochondrial fragmentation in these TNBC cells.

To test whether cystine-starvation-induced mitochondrial fragmentation is downstream of RIP1 activation, we treated the TNBC cells with the RIP1 inhibitor Nec-1 and found that Nec-1 might prevent the cystine-starvation-induced mitochondrial fragmentation (Figure 3C). Moreover, cystine starvation reduced the phosphorylation at serine 637 of DRP1 (Figure 3D). These results suggest that the cystine-starvation-induced mitochondrial fragmentation is downstream of RIP1 activation.

In addition, cystine starvation was found to decrease the basal oxygen consumption rate and the maximum respiratory rate (Figure 3E) and to increase mitochondrial ROS levels (Figure 3F). These results suggest that cystine starvation induces mitochondrial dysfunction and enhances mitochondrial ROS production. To evaluate whether the increase in mitochondrial ROS is important for cystine-starvation-induced cell death, we co-treated the TNBC cells with the mitochondrial ROS scavenger Necrox-5 and found that Necrox-5 can prevent cystine-starvation-induced cell death (Figure 3G). These results suggest that cystine starvation induces mitochondrial fragmentation, dysfunction, and ROS production during necroptosis. The increase in mitochondrial ROS production might contribute to cystine-starvation-induced cell death.

### The integrated stress response pathway is involved in the cystine-starvation-induced cell death

**Figure 4:**
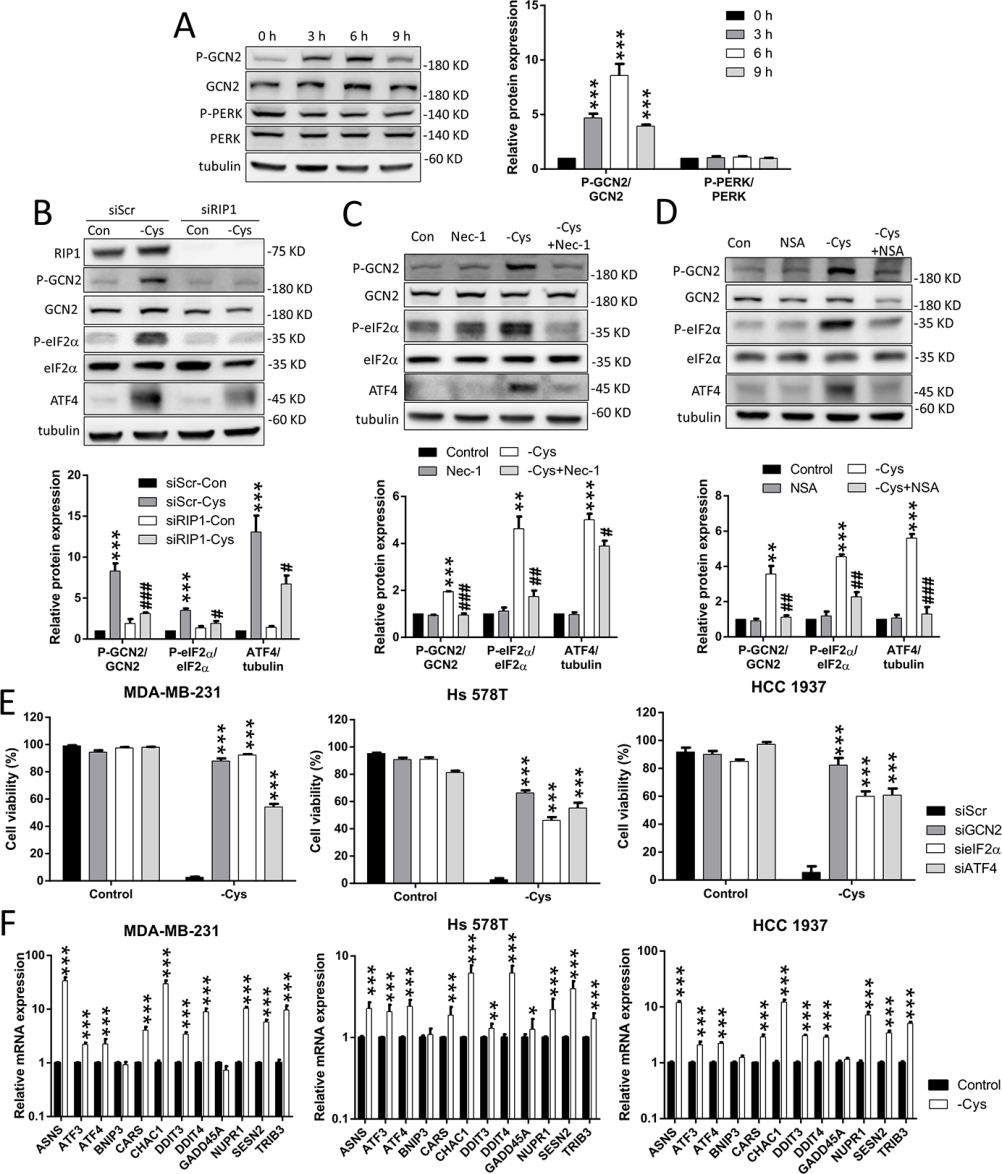
The GCN2-eIF2α-ATF4 pathway is involved in cystine-starvation-induced cell death (**A**) MDA-MB-231 cells were treated with cystine starvation for 3, 6, and 9 h. The phosphorylated GCN2 (threonine 899) and PERK (threonine 981) protein levels as well as the total GCN2 and PERK protein levels were determined by Western blot. (**B**) The RIP1 knockdown cells (MDA-MB-231) were treated with cystine starvation for 9 h. The phosphorylated GCN2 (threonine 899) and eIF2α (serine 51), RIP1, GCN2, eIF2α, and ATF4 protein levels were determined using Western blotting. (**C**, **D**) MDA-MB-231 cells were treated with cystine starvation with or without 16 μM Nec-1 (C) or 20 μM NSA (D) for 9 h. The phosphorylated GCN2 (threonine 899) and eIF2α (serine 51), GCN2, eIF2α, and ATF4 protein levels were determined using Western blotting. (**E**) The GCN2, eIF2α, or ATF4 knockdown cells (MDA-MB-231, Hs 578T, or HCC 1937) were treated with cystine starvation for 48 h. Cell viability was determined using the trypan blue exclusion assay. The knockdown efficiency of GCN2, eIF2α, and ATF4 are shown in Supplementary Figure 2. (**F**) MDA-MB-231, Hs 578T, and HCC 1937 cells were treated with cystine starvation for 9 h. The gene expression levels were detected using real-time PCR. Data represent the mean ± SEM of three independent experiments. ^*^p < 0.05, ^**^p < 0.01, ^***^p < 0.001 compared to the control group; ^#^*p* < 0.05, ^##^*p* < 0.01, ^###^*p* < 0.001 compared to the cystine starvation group. Con, control; -Cys, cystine starvation; Nec-1, necrostatin-1; NSA, necrosulfonamide; siScr, scramble.

To evaluate whether the integrated stress response is activated by cystine starvation, we examined the phosphorylation status of eIF2α and two of its upstream kinases: the general control nonderepressible 2 (GCN2) and the RNA-activated protein kinase-like ER kinase (PERK). We found that the phosphorylation level of GCN2 is increased in a time-dependent manner by cystine starvation, but the phosphorylation level of PERK is not (Figure 4A). Moreover, the phosphorylation levels of the eIF2α and ATF4 proteins are increased by cystine starvation. These results indicate that cystine starvation activates the integrated stress response in these TNBC cells.

To examine whether the activated GCN2-eIF2α-ATF4 pathway is downstream of RIP1-RIP3-MLKL activation, we treated the TNBC cells with siRNA against RIP1 and the specific inhibitors Nec-1 and NSA under cystine starvation. The results revealed that cystine starvation increases the phosphorylation levels of GCN2 and eIF2α, and that the elevated ATF4 expression is significantly inhibited by RIP1 knockdown, Nec-1 and NSA treatment (Figure 4B, 4C, 4D, Supplementary Figure 3A, 3B, and 3C). These results suggest that activation of the integrated stress response pathway is downstream of RIP1 and MLKL.

To determine whether the GCN2-eIF2α-ATF4 pathway contributes to cystine-starvation-induced cell death, we used siRNAs to knock down GCN2, eIF2α, and ATF4 in the TNBC cells. We found that the knockdown of GCN2, eIF2α, and ATF4 could significantly suppress cystine-starvation-induced cell death (Figure 4E). In addition, we analyzed the ATF4-regulated genes [29] and found that cystine starvation dramatically induced the expression of ATF4-regulated genes such as Asparagine Synthetase (ASNS), Activating Transcription Factor 3 (ATF3), Activating Transcription Factor 4 (ATF4), Cysteinyl-tRNA Synthetase (CARS), Glutathione-Specific Gamma-Glutamylcyclotransferase 1 (CHAC1), Sestrin 2 (SESN2), and Tribbles Pseudokinase 3 (TRIB3) (Figure 4F). These results suggest that the GCN2-eIF2α-ATF4 pathway is downstream of cystine-starvation-activated RIP1 and MLKL, and that ATF4-regulated genes might be involved in cystine-starvation-induced cell death.

### Cystine starvation induces CHAC1 expression and GSH degradation

**Figure 5:**
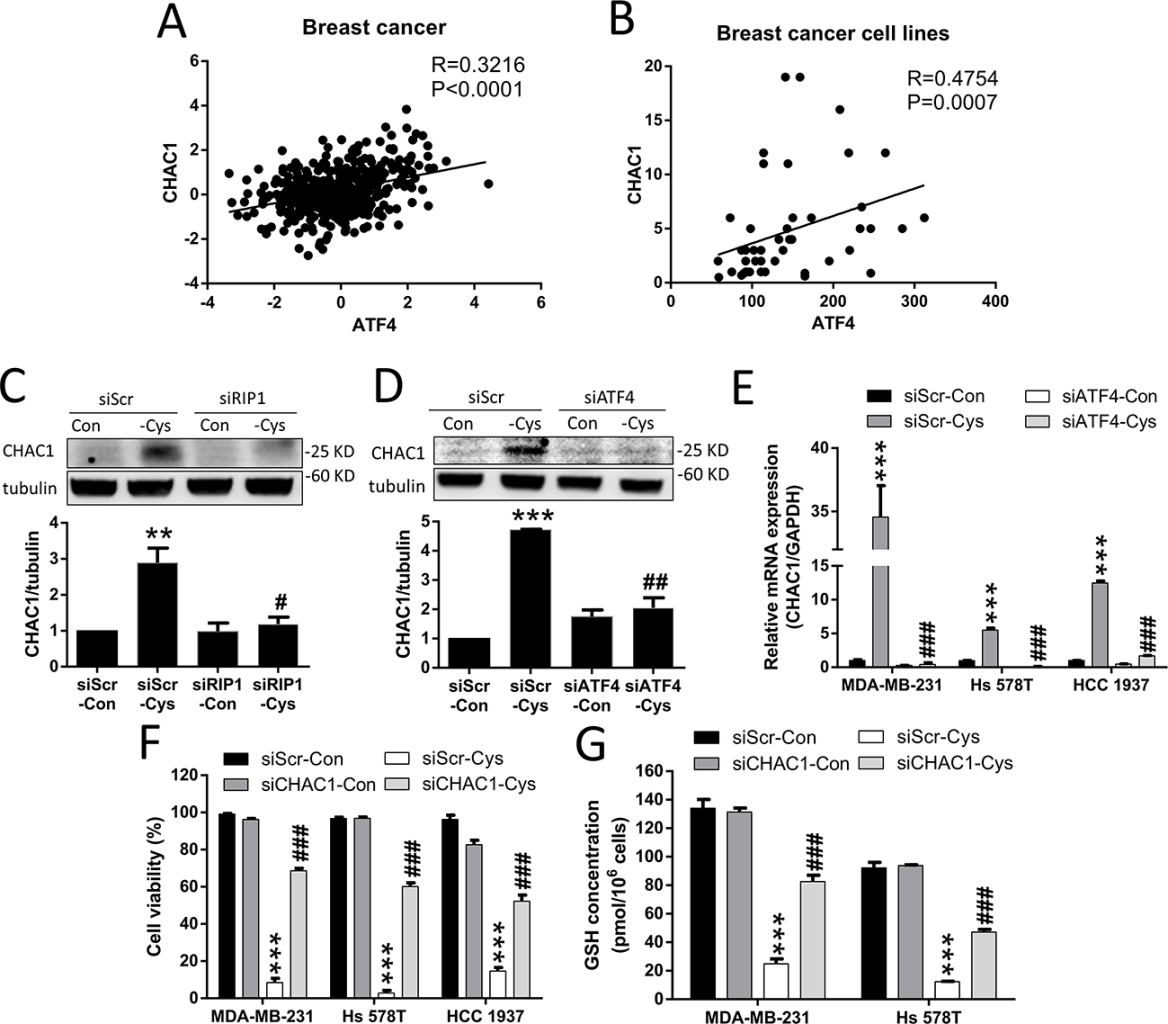
Cystine starvation induces CHAC1 expression and GSH degradation (**A**, **B**) Analysis of the correlation between ATF4 and CHAC1 expression using the open website database for breast cancer patients and cancer cells. (**C**, **D**) The MDA-MB-231 cells with RIP1 or ATF4 knockdown were treated with cystine starvation for 9 h. The CHAC1 protein levels were determined using Western blotting. (**E**) The MDA-MB-231, Hs 578T, and HCC 1937 cells with ATF4 knockdown were treated with cystine starvation for 9 h. The gene expression of CHAC1 was determined using real-time PCR. (**F**) The MDA-MB-231, Hs 578T, and HCC 1937 cells with CHAC1 knockdown were treated with cystine starvation for 48 h. Cell viability was determined using the trypan blue exclusion assay. The knockdown efficiency of CHAC1 is shown in Supplementary Figure 2. (**G**) The MDA-MB-231 and Hs 578T cells with CHAC1 knockdown were treated with cystine starvation for 24 h. The total GSH concentration was detected using a GSH assay kit. Data represent the mean ± SEM of three independent experiments. ^*^p < 0.05, ^**^p < 0.01, ^***^p < 0.001 compared to the control group; ^#^*p* < 0.05, ^##^*p* < 0.01, ^###^*p* < 0.001 compared to the cystine starvation group. Con, control; -Cys, cystine starvation; siScr, scramble.

CHAC1 was identified to have the function of digesting glutathione into 5-oxoproline and Cys-Gly dipeptide, thus decreasing intracellular GSH levels [30–32]. We found that CHAC1 is one of the largest changes in the gene expression induced by cystine starvation in the three TNBC cells (Figure 4F), and that there is a significant positive correlation between ATF4 and CHAC1 gene expression in breast cancer patients and breast cancer cell lines (Figure 5A and 5B). We thus hypothesized that upregulated CHAC1 might be required for cystine-starvation-induced cell death through GSH degradation.

To test our hypothesis, we first evaluated whether the increase in CHAC1 expression is downstream of the activated RIP1-ISR axis. Using siRNAs against RIP1 and ATF4, we found that cystine starvation-increased CHAC1 protein levels are inhibited by RIP1 knockdown (Figure 5C), and that the cystine-starvation-induced gene and protein expression of CHAC1 is inhibited by ATF4 knockdown (Figure 5D and 5E). These results suggest that the increased CHAC1 expression is downstream of the cystine starvation-activated RIP1-RIP3-MLKL-GCN2-eIF2α-ATF4 pathway in these TNBC cells.

Using siRNA against CHAC1, we demonstrated that the knockdown of CHAC1 is able to significantly suppress cystine-starvation-induced cell death (Figure 5F). In addition, the decrease in intracellular GSH levels induced by cystine starvation was significantly prevented by CHAC1 knockdown (Figure 5G). These results suggest that CHAC1 degradation of GSH might enhance cystine-starvation-induced cell death.

### High levels of ROS contribute to cystine-starvation-induced cell death

**Figure 6:**
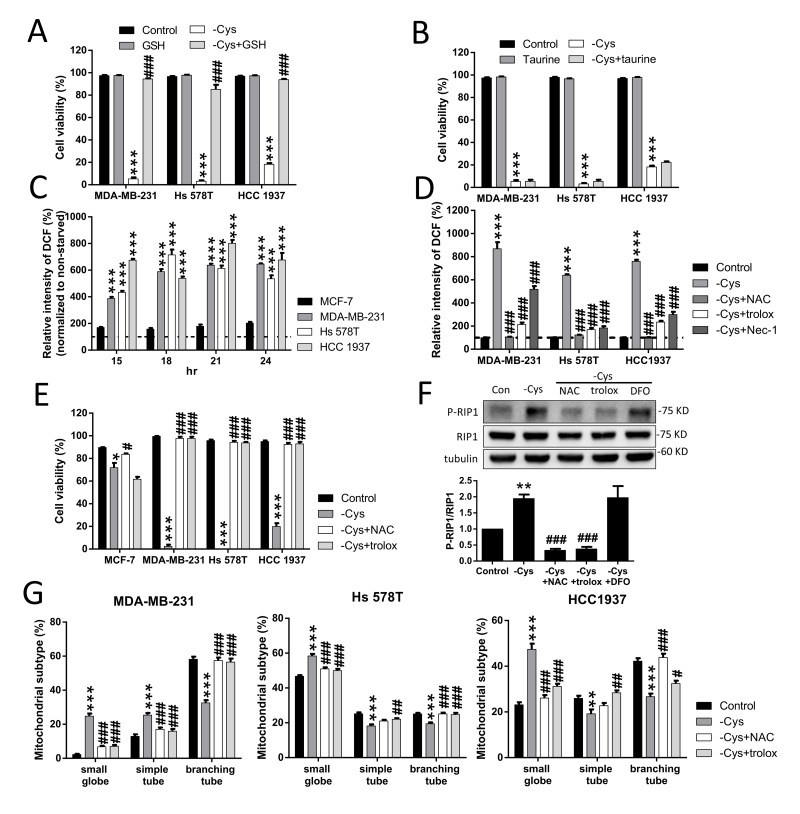
High levels of ROS contribute to cystine-starvation-induced cell death (**A**, **B**) MDA-MB-231, Hs 578T, and HCC 1937 cells were treated with cystine starvation with or without 50 μM GSH (A) or 50 μM taurine (B) for 48 h. Cell viability was determined using the trypan blue exclusion assay. (**C**) MCF-7, MDA-MB-231, Hs 578T, and HCC 1937 cells were treated with cystine starvation for 15, 18, 21, and 24 h. The levels of intracellular ROS were determined using flow cytometry with DCF staining. ^***^p < 0.001 compared to MCF-7 cells. (**D**) MDA-MB-231, Hs 578T, and HCC 1937 cells were treated with cystine starvation with or without 1 mM NAC, 100 μM Trolox, and 16 μM Nec-1 for 24 h. The levels of intracellular ROS were determined using flow cytometry with DCF staining. (**E**) MCF-7, MDA-MB-231, Hs 578T, and HCC 1937 cells were treated with cystine starvation with or without 1 mM NAC or 100 μM Trolox for 48 h. Cell viability was determined using the trypan blue exclusion assay. (**F**) Hs 578T cells were treated with cystine starvation with or without 1 mM NAC, 100 μM Trolox, and 100 μM DFO for 9 h. The P-RIP1 and RIP1 protein levels were determined using Western blotting. (**G**) MDA-MB-231, Hs 578T, and HCC 1937 cells were treated with cystine starvation with or without 1 mM NAC and 100 μM Trolox for 24 h. The mitochondrial morphology was determined using fluorescence microscopy with MitoTracker Green staining, and the mitochondrial morphology subtypes were classified by MicroP software. Data represent the mean ± SEM of three independent experiments. ^*^p < 0.05, ^**^p < 0.01, ^***^p < 0.001 compared to the control group; ^#^*p* < 0.05, ^##^*p* < 0.01, ^###^*p* < 0.001 compared to the cystine starvation group. Con, control; -Cys, cystine starvation; GSH, glutathione; NAC, N-acetyl-cysteine; Nec-1, necrostatin-1; DFO, deferoxamine.

Cystine is transported into the cytosol by the Xc- cystine/glutamate antiporter and reduced to cysteine as the sulfate donor for either GSH or taurine biosynthesis [33]. To evaluate whether GSH or taurine is important for cystine-starvation-induced cell death, we treated the TNBC cells with either GSH or taurine. We found that treatment with GSH can prevent cystine-starvation-induced cell death but treatment with taurine cannot (Figure 6A and 6B). These results suggest that a decrease in intracellular GSH biosynthesis induced by cystine starvation might result in cystine-starvation-induced cell death.

In addition, we found that after cystine starvation, the intracellular ROS levels are significantly increased by more than 400% in the TNBC cells and are higher than those in MCF-7 cells (Figure 6C). The increased ROS levels were inhibited by N-acetyl-L-cysteine (NAC), Trolox, and Nec-1 (Figure 6D). Furthermore, cystine-starvation-induced cell death (Figure 6E and Supplementary Figure 1A), RIP1 phosphorylation (Figure 6F), and mitochondrial fragmentation (Figure 6G) were suppressed by NAC and Trolox. These results suggest that high levels of intracellular ROS contribute to cystine-starvation-induced cell death.

## DISCUSSION

In this study, we demonstrated that the examined TNBC cells are highly susceptible to cystine starvation. Moreover, we found that cystine starvation can induce programed necrosis but not apoptosis or autophagy-mediated cell death in the TNBC cells. Mechanically, cystine starvation blocks the cysteine source for GSH biosynthesis and increases intracellular ROS levels, which trigger RIP1/RIP3/MLKL activation, mitochondrial damage, and mitochondrial ROS production. The activation of the GCN2-eIF2α-ATF4-CHAC1 pathway mediates GSH degradation, which further depletes intracellular GSH and results in enhanced, highly oxidative stress and cell death during cystine starvation.

Among the twenty amino acids, we found that cystine is the most essential amino acid for TNBC cell growth, and cystine starvation induces TNBC cell death. Cysteine is the reduced form of cystine, and cysteine/cystine is traditionally viewed as a nutritionally non-essential amino acid for humans. Human tissues, primarily the liver, can synthesize cysteine from L-methionine through the transsulfuration pathway [34]. Certain cancer cells cannot generate enough cysteine/cystine and thus depend on extracellular sources for growth and survival [34]. The Xc- cystine/glutamate antiporter was found to be expressed in one-third of TNBC tumors *in vivo* [13]. The plasma membrane antiporter mediates the cellular uptake of cystine from the environment. Inhibition of the Xc- cystine/glutamate antiporter [9, 13, 22, 23] and the systemic depletion of cystine with cystinase [35] were recently demonstrated to suppress breast tumor growth. Therefore, TNBC cells might highly depend on an extracellular source of cystine for cell survival and proliferation. These findings strongly suggest that inhibition of the Xc- transporter and/or cystine starvation may be potent anti-cancer strategies for TNBC.

In previous studies, inhibition of the Xc- transporter was found to induce non-apoptotic, iron-dependent, oxidative death (ferroptosis) [24, 36]. In the present study, we found that cystine starvation induces non-apoptotic cell death (Figures 1 and 2), and treatment with DFO, ferrostatin-1 (Figure 1H and Supplementary Figure 1) or antioxidants (Figure 5E) can prevent the cystine-starvation-induced cell death, suggesting that cystine starvation might induce ferroptosis in TNBC cells. Our results are consistent with the previous findings in MEF cells [24]. In addition to ferroptosis, we further demonstrated that cystine starvation can induce RIP1/RIP3/MLKL-dependent necrosis (Figure 1). Interestingly, DFO treatment was not able to prevent the cystine-starvation-induced activation of RIP1 (Figure 6F), suggesting that ferroptosis might be downstream of cystine-starvation-induced necroptosis in TNBC cells. Recent evidence revealed that cystine deprivation triggers RIP1/RIP3/MLKL-mediated necrosis in VHL-deficient renal cell carcinomas [9]. The molecular mechanism by which these TNBC cells are highly susceptible to cystine starvation needs further investigation.

In the cytosol, cystine is rapidly reduced to cysteine, which is required for protein synthesis, GSH biosynthesis and taurine synthesis and is a sulfate donor in mammalian cells [33]. In this study, we found that GSH, but not taurine, rescues cystine-starvation-induced cell death, suggesting that environmental cystine is essential for providing the cystine/cysteine needed for GSH synthesis in TNBC cells. GSH is a thiol-containing tripeptide consisting of glutamate, cysteine, and glycine that plays a critical role in cellular defenses against oxidative stress and toxic compounds [37]. We found that cystine starvation induces high levels of ROS (Figure 6). In addition, treatment with antioxidants NAC and Trolox can prevent ROS production and cell death in TNBC cells in response to cystine starvation (Figure 6). These findings suggest that cystine starvation induced a reduction in GSH synthesis and an increase in ROS production that contribute to necroptosis and ferroptosis in TNBC cells.

**Figure 7:**
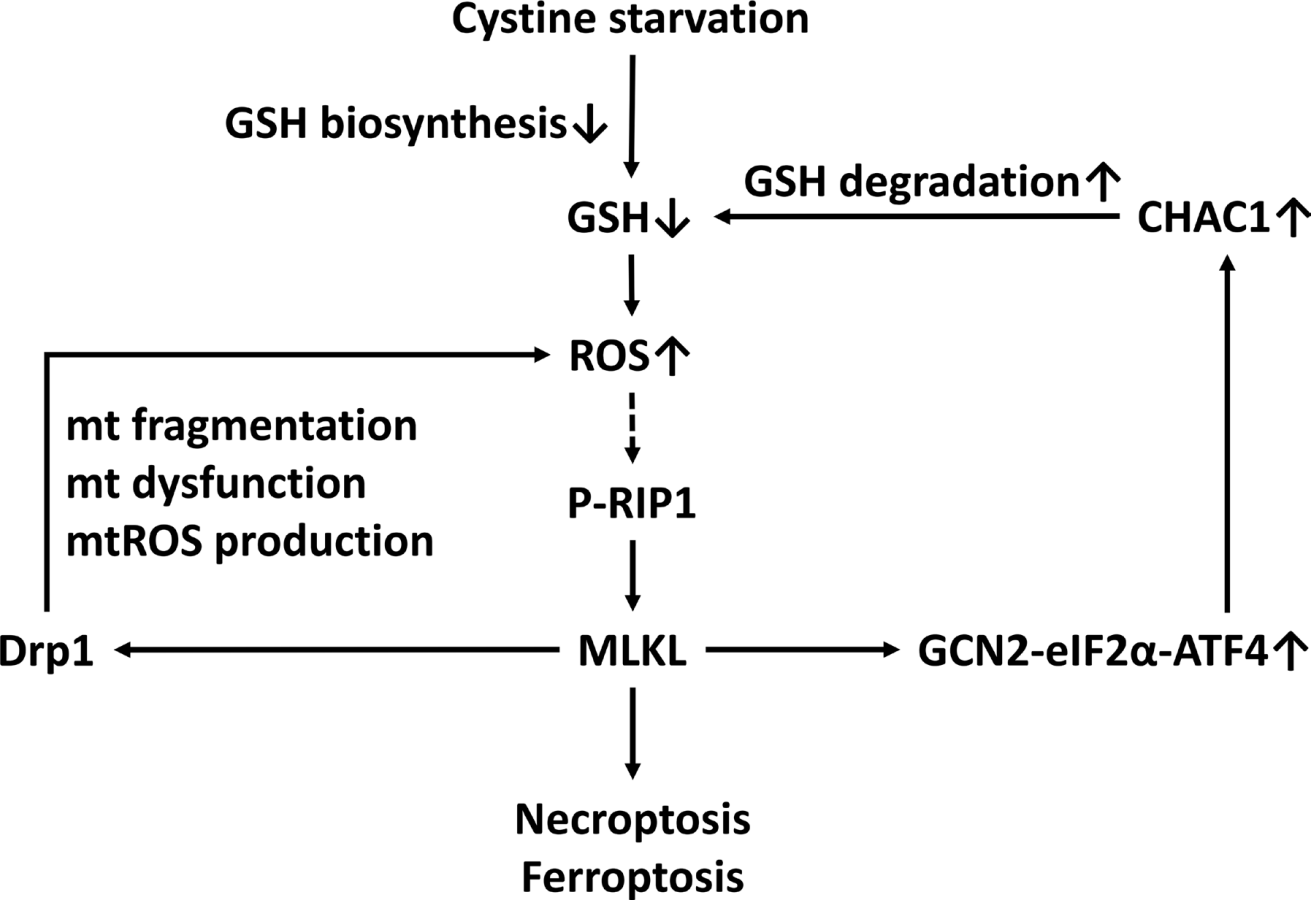
A scheme of the mechanism of CHAC1 degradation of glutathione enhancing cystine-starvation-induced necroptosis and ferroptosis in human triple negative breast cancer cells via the GCN2-eIF2α-ATF4 pathway

Although the integrated stress response is important for cellular survival and homeostasis in response to various types of stress, exposure to severe stress can drive signaling toward cell death [21]. The core event in the integrated stress response pathway is the phosphorylation of eIF2α at serine 51 by one of the four eIF2α kinases: GCN2, PERK, dsRNA-activated protein kinase R (PKR), and heme-regulated inhibitor eIF2α kinase (HRI) [21]. GCN2 and PERK are the most common eIF2α kinases that are activated by amino acid starvation [21, 38]. It was established that the phosphorylation of eIF2α attenuates the initiation of global cap-dependent protein translation but concurrently allows the translation of selected genes with small upstream open reading frames, including the ATF4 transcription factor, and that ATF4 target genes contribute to the cellular adaption to stressors. The gene expression program activated by the integrated stress response optimizes the cellular response to stress and is dependent on the intensity of the stress stimuli [21, 39]. Several lines of evidence indicated that the GCN2-eIF2α-ATF4 pathway contributes to apoptosis under metabolic stress [18, 40–42]. In this study, we demonstrated for the first time that cystine starvation can activate GCN2, but not PERK, to increase the phosphorylation of eIF2α at serine 51, the protein expression of ATF4, and the gene expression of ATF4 target genes such as CHAC1 (Figure 4). Moreover, the activated integrated stress response pathway was demonstrated to be downstream of the RIP1/RIP3-MLKL pathway and to contribute to cystine-starvation-induced necroptosis (Figure 4). In addition, upregulated CHAC1 might degrade intracellular GSH, and the intracellular GSH pool might thus be rapidly depleted by both cystine starvation and CHAC1 activation. Therefore, both the decrease in GSH synthesis by cystine starvation and the increase in GSH degradation by CHAC1 expression may result in high levels of oxidative stress and necroptosis/ferroptosis (Figure 7).

In conclusion, we demonstrated for the first time that the GCN2-eIF2α-ATF4 pathway enhances cystine-starvation-induced necroptosis and ferroptosis through CHAC1 degradation of GSH and high levels of oxidant stress in human TNBC cells. Our findings might provide a mechanism for cystine-starvation-induced programed necrosis in TNBC cells.

## MATERIALS AND METHODS

### Cell culture and amino acid starvation

The human breast cancer cell lines MCF-7, MDA-MB-231, and Hs 578T were cultured in DMEM (United States Biological) with 10% fetal bovine serum (FBS, Life Technologies), 1% penicillin/streptomycin (P/S), and 1% non-essential amino acids at 37°C and 5% CO_2_ in an incubator. The human breast cancer cell line HCC 1937 was cultured in RPMI 1640 (United States Biological) with 10% FBS and 1% P/S. To prepare the culture medium for each amino acid starvation condition, DMEM or RPMI 1640 medium without amino acids (D9800-13 and R9010-01, United States Biological) was mixed with different amino acid combinations lacking a specific amino acid (LAA21-1KT, Sigma-Aldrich).

### Cell growth and viability assay

To screen for amino acids that are highly required for TNBC cell growth, cells were seeded in a 96-well dish at a density of 3,000 cells per well with amino acid starvation medium. After 48 h, the cells were fixed with 10% trichloroacetic acid (TCA, Sigma-Aldrich). After the cells were washed with distilled water, they were stained with sulforhodamine B (SRB, Sigma-Aldrich) and washed with 1% acetic acid. The stained cells were assessed by absorbance at 510 nm using a microplate reader. For cell viability, cells were seeded in a 6-well culture dish at a density of 3 × 10^5^ cells per well. The dead and live cell suspensions were counted using a hemocytometer after 0.4% trypan blue (Sigma-Aldrich) staining.

### Small interfering RNA (siRNA)-mediated specific gene knockdown

Cells were seeded in a 60-mm dish at a density of 6 × 10^5^ cells. Lipofectamine RNAi MAX reagent (Invitrogen^TM^, Thermo Scientific-Technologies) was incubated with 60 pmol siRNA in OPTI-MEM medium (Gibco^TM^, Thermo Scientific-Technologies) for 5 min at room temperature. The siRNA-reagent mixture was added to the culture medium and incubated for 48 h for further experiments. The specific ON-TARGET plus^TM^ SMARTpool RIP1 (L-004445), EIF2AK4 (GCN2, L-005314), EIF2S1 (eIF2α, L-015389), ATF4 (L-004445), CHAC1 (L-004445) and non-target (scramble, D-001810) siRNAs used in this study were purchased from GE Healthcare Dharmacon (Lafayette Co., USA).

### Western blot

Cell lysate from the 60 mm dishes was extracted using radioimmunoprecipitation assay buffer (RIPA buffer: 50 mM Tris-HCl buffer (pH 7.5) containing 0.15 M NaCl, 0.5% sodium deoxycholate, 0.5% SDS, and 0.1% Triton X-100) and 10 μg/ml aprotinin, 2 mM EDTA, 2 mM Na_3_VO_4_, and 1 mM PMSF. The protein concentration of the cell lysate was determined using the Bradford reagent with bovine serum albumin (BSA) as the standard (Sigma-Aldrich). In total, 20 μg of lysate protein was resolved by SDS-polyacrylamide gel electrophoresis and then transferred onto PVDF membranes. The membranes were blocked for an hour at room temperature with a 5% non-fat milk solution in TBST buffer (20 mM Tris-HCl (pH 7.6), 0.137 M NaCl, and 1% Tween-20). The blots were incubated with primary and secondary antibodies overnight. The blots were then washed three times in TBST. The images of the Western blots were observed by the FUJIFILM LAS-4000 system.

### Transmission electron microscopy

Cells were seeded in a 60-mm dish at a density of 6 × 10^5^ cells. After cystine starvation for 24 h, the cells were fixed with 2% glutaraldehyde and 3% paraformaldehyde in 0.1 M sodium cacodylate buffer for 1 h, fixed in 1% osmium tetroxide for 1.5 h, and further incubated with 1% uranyl acetate for 1 h. Then, the samples were dehydrated in ethanol and embedded in Epon-Araldite embedding resin. Ultrathin sections were collected on formvar-coated grids and stained with uranyl acetate for 10 min and then with lead citrate for 5 min. The samples were observed with a JEM-2000EXII transmission electron microscope.

### Fluorescence microscopy

Cells were seeded in a 6-well dish at a density of 3 × 10^5^ cells per well. After cystine starvation for 24 h, the cells were washed twice with PBS after staining with 250 μM MitoTracker Green (Life Technologies) for 30 min. Mitochondrial morphology was determined with a fluorescence microscope (OLYMPUS IX70), and the subtype of mitochondria was classified by MicroP software [43].

### Intracellular ROS and mitochondrial ROS

DCFH-dA was used to determine the intracellular ROS levels. After incubation with 10 μM DCFH-dA for 30 min, cells were washed with PBS and resuspended in PBS. The DCF fluorescence intensity at FL1 was determined by FACSCalibur (Becton Dickinson, Bedford, MA, USA). For mitochondrial ROS, after incubation with 10 μM MitoSOX Red for 20 min, cells were washed with PBS and resuspended in PBS. The MitoSOX Red fluorescence intensity at FL2 was determined by FACSCalibur (Becton Dickinson, Bedford, MA, USA).

### Oxygen consumption rate

Oxygen consumption rates were determined using an XF24 Extracellular Flux Analyzer (Seahorse Bioscience, North Billerica, MA, USA). Cells were seeded in 24-well plates at a density of 3 × 10^4^ cells per well with cystine starvation for 24 and 48 h. The culture medium was replaced with the assay medium containing sodium carbonate-free DMEM (pH 7.4). Prior to the assay, the cell plate and sensor cartridge were incubated in 1 ml of Seahorse Bioscience XF-24 Calibrant per well in a Seahorse Bioscience 24-well plate at 37°C without CO_2_ overnight. The basal, proton-leaked, maximal, and non-mitochondrial OCRs were sequentially measured before and after the injection of 2 μg/ml oligomycin, 5 μg/ml carbonyl cyanide-p-trifluoromethoxyphenylhydrazone (FCCP), and 5 μg/ml antimycin A (Sigma-Aldrich), respectively. The Seahorse XF-24 Analyzer program was set according to the manufacturer’s instructions.

### Intracellular glutathione

Cells were seeded in a 60-mm dish (Corning Inc., Corning, NY, USA) at a density of 6 × 10^5^ cells for cystine starvation. The cells were lysed with 5% 5-sulfosalicylic acid solution. The cellular level of glutathione was determined using a Glutathione Assay Kit (Sigma-Aldrich, St. Louis, MO, USA) according to the manufacturer’s protocol.

### Real-time PCR

Total RNA was extracted using TRIzol reagent (Invitrogen^TM^, Thermo Scientific-Technologies). Reverse transcript reactions used total RNA (5 μg) obtained by RevertAid^TM^ reverse transcriptase (Thermo Fisher Scientific). Real-time PCR amplification was performed by KAPA SYBR^TM^ FAST qPCR Kits with Applied Biosystems^TM^ Real-Time PCR Instruments. Amplification of cDNA started with 3 minutes at 95°C, followed by 50 cycles of 3 seconds at 95°C and 30 seconds at 60°C. The primer sequences are presented in Supplementary Table 1.

### Bioinformatics analyses

Correlations of gene expression in breast cancer patients and breast cancer cells were determined in clinical microarrays existing at cBioPortal (http://www.cbioportal.org/) [44, 45] and the European Bioinformatics Institute (EMBL-EBI) (https://www.ebi.ac.uk). The data set from cBioPortal was considered The Cancer Genome Atlas (TCGA) database [46]. Data were processed and analyzed with GraphPad PRISM 6.

### Statistical analysis

All of the data are presented as the mean ± SEM. GraphPad PRISM software version 6 (GraphPad Software) was used for all statistical analyses. The statistical significance of the differences between two groups was calculated using an unpaired Student’s *t*-test. A *p* value <0.05 was considered to be statistically significant.

## SUPPLEMENTARY MATERIALS FIGURES AND TABLE


