# Supplementary material for: CHAC1 degradation of glutathione enhances cystine-starvation-induced necroptosis and ferroptosis in human triple negative breast cancer cells via the GCN2-eIF2α-ATF4 pathway
Source: Oncotarget. 2017 Dec 9;8(70):114588–602. doi: 10.18632/oncotarget.23055 (PMC5777716; doi:10.18632/oncotarget.23055)
Supplement: Supplementary file 1 [file oncotarget-08-114588-s001.pdf]

CHAC1 degradation of glutathione enhances cystine-starvation-induced necroptosis and ferroptosis in human triple negative breast cancer cells via the GCN2-eIF2α-ATF4 pathway

SUPPLEMENTARY MATERIALS

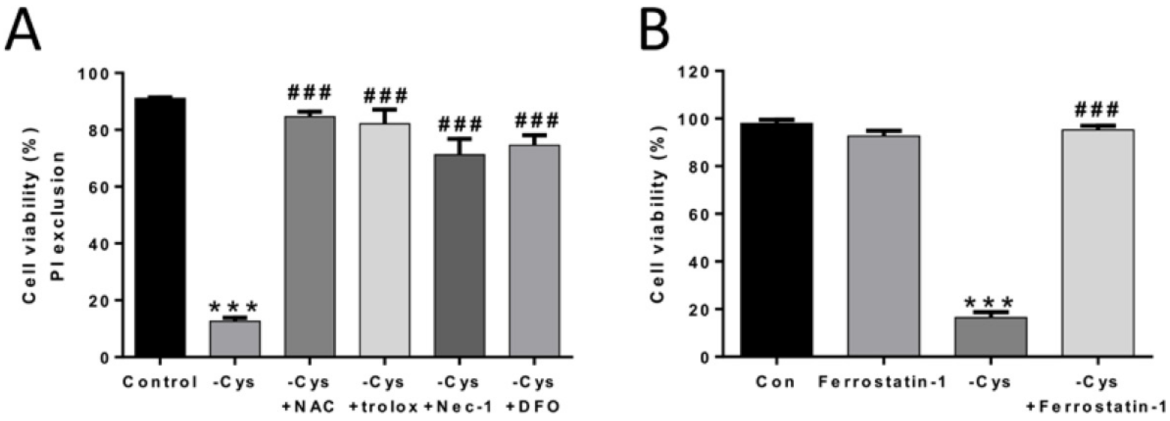

Supplementary Figure 1: Effects of antioxidants (NAC and trolox), Nec-1, DFO, ferrostatin-1 on the cystine-starvation-induced cell death. (**A**) Hs 578T cells were treated with cystine starvation with or without 1 mM NAC, 100 µM trolox, 16 µM Nec-1, or 100 µM DFO for 48 h. The cell viability was determined using the flow cytometry with propidium iodide (PI) staining. (**B**) HCC 1937 cells were treated with cystine starvation with or without 20 µM ferrostatin-1 for 48 h. Cell viability was determined using trypan blue exclusion assay. Data represent the mean ± SEM of three independent experiments. $^{*}p < 0.05$, $^{**}p < 0.01$, $^{***}p < 0.001$ compared to the control group; $^{\#}p < 0.05$, $^{\#\#}p < 0.01$, $^{\#\#\#}p < 0.001$ compared to the cystine starvation group. Con, control; -Cys, cystine starvation; NAC, N-acetyl-cysteine; Nec-1, necrostatin-1; DFO, deferoxamine.

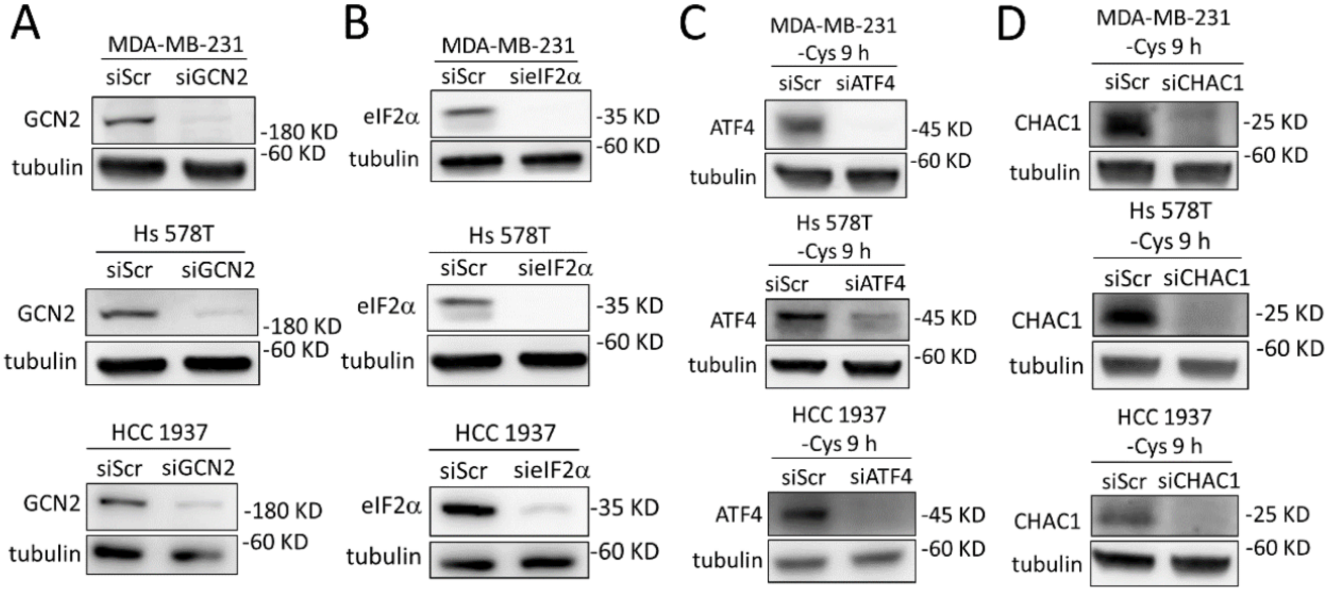

Supplementary Figure 2: Knockdown efficiency of the siRNAs against GNC2, eIF2α, ATF4, or CHAC1 in TNBC cells. (**A**, **B**) After treatments with specific siRNAs against GCN2 or eIF2α for 24 h, the protein levels of GCN2 (A) or eIF2α (B) were determined using Western blotting. (**C**, **D**) The MDA-MB-231, Hs 578T, and HCC 1937 cells with ATF4 or CHAC1 knockdown were treated with cystine starvation for 9 h. The protein levels of ATF4 (C) and CHAC1 (D) were determined using Western blotting.

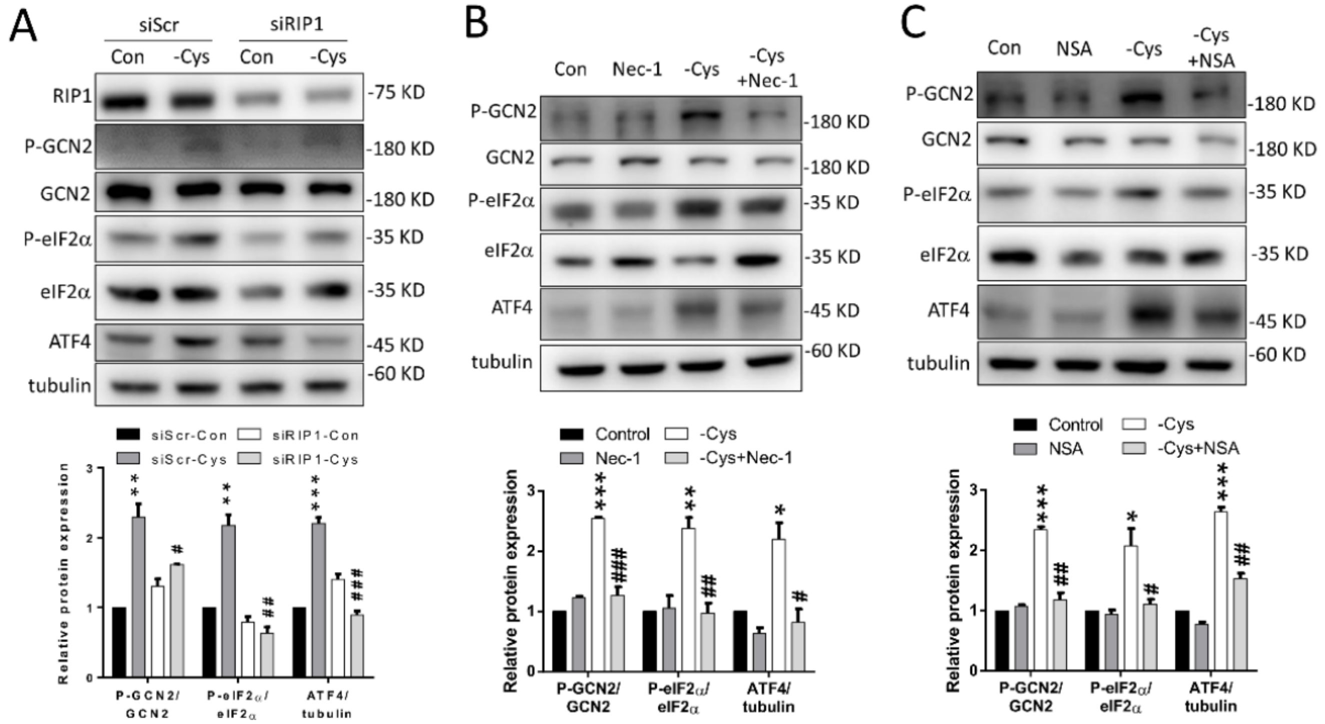

Supplementary Figure 3: The GCN2-eIF2α-ATF4 pathway is regulated by RIP1/RIP3/MLKL in HCC 1937 cells. (**A**) The RIP1 knockdown cells (HCC 1937) were treated with cystine starvation for 9 h. The phosphorylated GCN2 (threonine 899) and eIF2α (serine 51), RIP1, GCN2, eIF2α, and ATF4 protein levels were determined using Western blotting. (**B**, **C**) HCC 1937 cells were treated with cystine starvation with or without 16 μM Nec-1 (B) or 20 μM NSA (C) for 9 h. The phosphorylated GCN2 (threonine 899) and eIF2α (serine 51), GCN2, eIF2α, and ATF4 protein levels were determined using Western blotting. Data represent the mean ± SEM of three independent experiments. $^{*}p < 0.05$, $^{**}p < 0.01$, $^{***}p < 0.001$ compared to the control group; $^{\#}p < 0.05$, $^{\#\#}p < 0.01$, $^{\#\#\#}p < 0.001$ compared to the cystine starvation group. siScr, scramble; Con, control; -Cys, cystine starvation; Nec-1, necrostatin-1; NSA, necrosulfonamide.

Supplementary Table 1: The primer sequences used in this study

Gene	Primer sequence (5′-to-3′)
ASNS-f	5′-CGACCAAAAGAAGCCTTCAG-3′
ASNS-r	5′-GCCATCATTGCATCATCAAC-3′
ATF3-f	5′-AAGAACGAGAAGCAGCATTTGAT-3′
ATF3-r	5′-TTCTGAGCCCGGACAATACAC-3′
ATF4-f	5′-TCAAACCTCATGGGTTCTCC-3′
ATF4-r	5′-GTGTCATCCAACGTGGTCAG-3′
BNIP3-f	5′-TCAGCATGAGGAACACGAGC-3′
BNIP3-r	5′-GAGGTTGTCAGACGCCTTCC-3′
CARS-f	5′-GCACAGCTCTTGGAGGATGT-3′
CARS-r	5′-GAGTCTGGACTGCACAGCTT-3′
CHAC1-f	5′-CCTGAAGTACCTGAATGTGCGAGA-3′
CHAC1-r	5′-GCAGCAAGTATTCAAGGTTGTGGC-3′
DDIT3-f	5′-TCCTGGAAATGAAGAGGAAGA-3′
DDIT3-r	5′-TGTGACCTCTGCTGGTTCTG-3′
DDIT4-f	5′-GTTTGACCGCTCCACGAGCCT-3′
DDIT4-r	5′-GCACACAAGTGTTCATCCTCAGG-3′
GADD45A-f	5′-GCTGGTGACGAATCCACATT-3′
GADD45A-r	5′-TCACTGGAACCCATTGATCC-3′
NUPR1-f	5′-AGAAGCTGCTGCCAACACCA-3′
NUPR1-r	5′-TAGTGTCCATGGTCTGGCCTC-3′
SESN2-f	5′-TTCGGATATGAGGACTTCAC-3′
SESN2-r	5′-ATGGTATTGTAGGTGAGGCT-3′
TRIB3-f	5′-CGTGAGAGGAAGAAGCTGGT-3′
TRIB3-r	5′-CTCAGGTCCCACGTAGGCT-3′
